# A Diffusion-Reaction Model for Predicting Enzyme-Mediated Dynamic Hydrogel Stiffening

**DOI:** 10.3390/gels5010017

**Published:** 2019-03-13

**Authors:** Hung-Yi Liu, Chien-Chi Lin

**Affiliations:** 1 Weldon School of Biomedical Engineering, Purdue University, West Lafayette, IN 47907, USA; liu1808@purdue.edu; 2 Department of Biomedical Engineering, Purdue School of Engineering & Technology, Indiana University-Purdue University Indianapolis, Indianapolis, IN 46202, USA

**Keywords:** diffusion-reaction, dynamic hydrogel, matrix stiffening, tyrosinase

## Abstract

Hydrogels with spatiotemporally tunable mechanical properties have been increasingly employed for studying the impact of tissue mechanics on cell fate processes. These dynamic hydrogels are particularly suitable for recapitulating the temporal stiffening of a tumor microenvironment. To this end, we have reported an enzyme-mediated stiffening hydrogel system where tyrosinase (*Tyr_ase_*) was used to stiffen orthogonally crosslinked cell-laden hydrogels. Herein, a mathematical model was proposed to describe enzyme diffusion and reaction within a highly swollen gel network, and to elucidate the critical factors affecting the degree of gel stiffening. Briefly, Fick’s second law of diffusion was used to predict enzyme diffusion in a swollen poly(ethylene glycol) (PEG)-peptide hydrogel, whereas the Michaelis–Menten model was employed for estimating the extent of enzyme-mediated secondary crosslinking. To experimentally validate model predictions, we designed a hydrogel system composed of 8-arm PEG-norbornene (PEG8NB) and bis-cysteine containing peptide crosslinker. Hydrogel was crosslinked in a channel slide that permitted one-dimensional diffusion of *Tyr_ase_*. Model predictions and experimental results suggested that an increasing network crosslinking during stiffening process did not significantly affect enzyme diffusion. Rather, diffusion path length and the time of enzyme incubation were more critical in determining the distribution of *Tyr_ase_* and the formation of additional crosslinks in the hydrogel network. Finally, we demonstrated that the enzyme-stiffened hydrogels exhibited elastic properties similar to other chemically crosslinked hydrogels. This study provides a better mechanistic understanding regarding the process of enzyme-mediated dynamic stiffening of hydrogels.

## 1. Introduction

Hydrogels are hydrophilic and crosslinked water-swollen polymers [1,2,3] particularly suitable for mimicking extracellular matrix (ECM) in human tissues [4,5]. The effects of ECM compositions and degradability on cell fate processes have been extensively studied [6,7]. In recent years, mechanical properties of the ECM are also deemed as a crucial factor regulating tissue regeneration and disease progression [8,9]. As such, hydrogels with spatiotemporally regulated mechanics are increasingly utilized for studying mechanotransduction in healthy and diseased cells. Mechanical properties of a water-swollen hydrogel have been described by Anseth and colleges using Flory–Rehner and rubber elasticity theories [10,11]. In general, hydrogel elasticity, viscosity, and plasticity are characterized via dynamic mechanical analysis or shear rheometry [12,13]. Mechanical properties of a swollen hydrogel are directly related to gel crosslinking density, which are determined by macromer functionality, precursor compositions, polymerization conditions, and degree of gel swelling [14]. Understanding the impact of these factors on hydrogel crosslinking is instrumental when preparing hydrogels with user-defined and highly tunable mechanical properties [15].

Hydrogels can be prepared through chemical reactions (e.g., chain-growth or step-growth polymerization) or physical interactions (e.g., electrostatic or supramolecular binding) [16,17]. Regardless of the crosslinking method, however, bulk modulus of a hydrogel generally scales with its crosslinking density [14,18]. Therefore, hydrogels with a built-in mechanism for post-gelation adjustment of crosslinking density are ideal for mimicking the stiffening process of a diseased tissue. In this regard, various dynamic cell-laden gel systems are being actively pursued for studying the changes of tissue mechanics on cell behaviors [19,20,21,22,23]. In principle, dynamic stiffening hydrogels are fabricated with a two-step crosslinking process. The first crosslinking technique forms a cell-laden gel network with additional polymerizable moieties for secondary polymerization [21,24,25]. The latter increases the crosslinking density and hence stiffness of the cell-laden hydrogel. For example, Young et al. designed cardiomyocyte-encapsulated hydrogels crosslinked by thiolated hyaluronic acid (THA) and poly(ethylene glycol)-diacrylate (PEGDA) [26]. The cell-laden hydrogels were stiffened gradually via thiol–acrylate Michael-type addition. While the two-step click reactions achieved a dynamic increase in gel crosslinking, the degree of stiffening was pre-determined by the thiol and acrylate contents, which could not be modularly and dynamically controlled post-gelation.

In order to control the degree of matrix stiffening, our lab has introduced an enzyme-mediated dynamic hydrogel system [27,28]. The primary cell-laden hydrogel network was formed by thiol–norbornene photochemistry using PEG-8-arm norbornene (PEG8NB) and peptide crosslinkers (i.e., CYGGGYC). Here, we incorporated additional tyrosine residues on the peptide crosslinker sequence to serve as substrates for tyrosinase (*Tyr_ase_*). Upon diffusing into the gel network, *Tyr_ase_* oxidizes peptidyl *Tyr* residues to generate 3,4-dihydroxy-phenylalanine (DOPA) that leads to the formation of additional DOPA dimer [29,30]. The latter was key to the dynamic gel stiffening scheme. Previously, we have applied the *Tyr_ase_*-stiffened cell-laden gels to evaluate the impact of dynamic matrix stiffening on activation of pancreatic stellate cells (PSCs) [28] and the metastatic potential of pancreatic cancer cells (PCCs) [27]. To provide a better understanding of enzyme diffusion and reaction during the gel stiffening process, we describe here a mathematical model that accounts for both enzyme diffusion and reaction within the highly swollen hydrogel network. The crucial parameters associated with Fick’s second law and Michaelis–Menten kinetics were investigated, including diffusion and catalytic reactions of *Tyr_ase_*. Furthermore, we employed a channel slide to experimentally validate the prediction of one-dimensional *Tyr_ase_* diffusion into the PEG-peptide hydrogel [31]. These experimental data were further used to verify and improve model predictions, which offer pivotal information when applying enzymatic reaction for dynamic stiffening of cell-laden hydrogels.

## 2. Results and Discussion

### 2.1. Design Principle of the Primary Hydrogel Network

**Figure 1:**
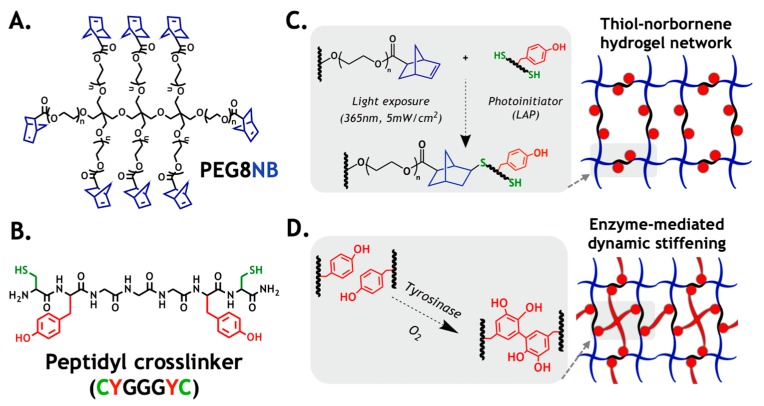
Design principle of the enzyme-mediated hydrogel stiffening: (**A**) structure of 8-arm PEG-norbornene (PEG8NB, 20 kDa, *n* ~ 56); (**B**) structure of an example peptide crosslinker (i.e., CYGGGYC); (**C**) schematic of thiol–norbornene photopolymerization to form primary hydrogel network; (**D**) schematic of enzyme (*Tyr_ase_*)-catalyzed DOPA dimer formation, which leads to dynamic hydrogel stiffening.

In this work, we used a bio-inert macromer PEG8NB (Figure 1A) and a bifunctional peptidyl crosslinker (CYGGGYC, Figure 1B) as an experimental model to verify the computational prediction of enzyme-induced matrix stiffening. The major advantage of a PEG-based gel prepared from orthogonal step-growth polymerization is its high gelation efficiency that produces a well-defined and near ideal network structure (Figure 1C). Additionally, thiol–norbornene hydrogel affords more uniform distribution of tyrosine residues in the primary gel network, which increases substrate accessibility for *Tyr_ase_* (Figure 1D). In principle, the infiltrating *Tyr_ase_* catalyzes DOPA dimer formation, which leads to an increased gel crosslinking density and modulus.

### 2.2. Correlation of Gel Crosslinking Density, Mesh Size, and Enzyme Diffusivity

Hydrogel crosslinking density has a significant impact on the diffusion of soluble molecules in the network [32]. More specifically, mesh size of a gel network is the primary factor determining diffusivity of any solute in a highly swollen hydrogel. During the process of enzyme-mediated gel stiffening, hydrogel crosslinking density increases not only with time, but may also vary spatially. Consequently, the diffusivity of *Tyr_ase_* may be impacted by the stiffening network. The diffusivity of any solute in a highly swollen hydrogel can be estimated by the classical Lustig–Peppas relationship, which describes solute diffusivity (i.e., *D_E_^gel^*) using a correlation of hydrodynamic radius of the solute (*R_E_*), mesh size of the network (*ξ*), and the diffusivity of the solute in a solution (see Section 3.3). Many of these parameters can be obtained from the literature or be determined experimentally. To gain insight into the impact of gel crosslinking on enzyme diffusion in hydrogels, we prepared hydrogels with different macromer compositions that led to varying shear moduli (G’ ~0.5 to ~10 kPa). At any given formulation (and modulus), the mass (*q*) and volumetric (*Q*) swelling ratio, as well as the corresponding mesh size (*ξ*), of the resulting hydrogels could be readily determined. These results were then used to establish a correlation between gel stiffness and *D_E_^gel^* (see Section 3.3). 

**Figure 2:**
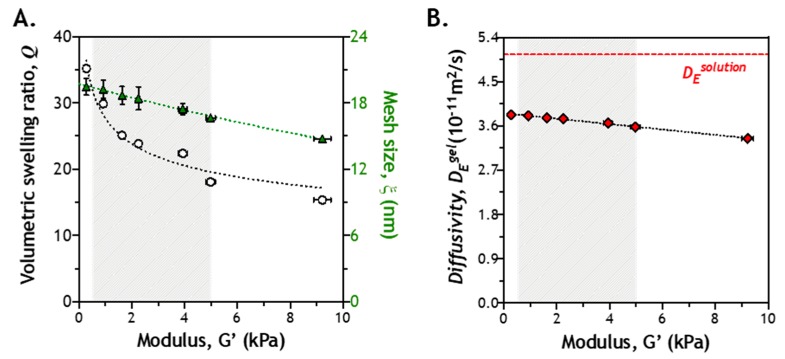
Correlation of crosslinking density, mesh size, and enzyme diffusivity: (**A**) volumetric swelling ratio (*Q*) and mesh size (*ξ*) of PEG-peptide (CYGGGYC) hydrogels with different shear moduli (*G’*); (**B**) correlation of enzyme diffusivity (*D_E_^gel^*) with gel modulus. *D_E_^solution^* is the diffusivity of enzyme (i.e., tyrosinase) in solution.

In an earlier work, we characterized the shear moduli of *Tyr_ase_*-mediated stiffening hydrogels, which varied from ~0.5 to ~5 kPa [28]. This range of gel stiffness is relevant to changes of tissue stiffness during tumor progression [33]. Therefore, we experimentally determined *Q* and *ξ* of hydrogels within shear moduli from ~0.5 to ~5 kPa. As polymer content increases, *Q* was decreased from ~30 to ~18, while *ξ* was correspondingly decreased from ~19 to ~15 nm (Figure 2A). It is important to note that this range of mesh size is much larger than the hydrodynamic radius of *Tyr_ase_* (*R_E_* = 4.5 nm) [34]. Next, we estimated *D_E_^gel^* using the Lustig–Peppas relationship [35]. Clearly, diffusivity of *Tyr_ase_* in solution (*D_E_^solution^*) was not significantly affected by changes of gel mesh size (i.e., both *ξ* and *Q* approach infinity). In a soft gel (e.g., G’ ~ 0.5 kPa), *D_E_^gel^* was 3.80 × 10^−11^ m^2^/s. In a stiff gel (e.g., G’ ~5 kPa), it was decreased slightly to 3.58 × 10^−11^ m^2^/s (Figure 2B). Since *D_E_^gel^* in a stiffer gel is only ~5.8% smaller than that in a softer gel, the gradually increasing gel crosslinking during the stiffening process should not impose a significant diffusion hindrance to *Tyr_ase_*.

### 2.3. Prediction of Enzyme Diffusion in Hydrogels with Different Crosslinking Density

Correlations of gel modulus, mesh size, and enzyme diffusivity as shown in Figure 2 have provided critical information regarding the extent to which *D_E_^gel^* was affected by an increasing gel crosslinking density. To establish the premise that the gradually stiffened hydrogel would not impose significant diffusion hindrance to *Tyr_ase_*, we predicted *Tyr_ase_* distribution (Equation (1)) within the hydrogels using a constant *D_E_^gel^*, which yields the following equation:

(1) $$\frac{\partial C_{E}(x,t)}{\partial t}=-D_{E}{}^{gel}\times \frac{\partial^{2}C_{E}(x,t)}{\partial x^{2}}$$

here, 3.80 × 10^−11^ m^2^/s and 3.58 × 10^−11^ m^2^/s were used to represent *D_E_^gel^* in a soft and stiff gel, respectively. If distributions of *Tyr_ase_* in hydrogels with these two diffusivities show negligible differences within a relevant time scale, it can be safely assumed that the stiffening network only exhibits a minimal hindrance on enzyme transport. Equation (1) was solved numerically using the initial and boundary conditions listed in Section 3.3 [36]:

(2) CE(x,t)=CE0⋅{1−4π∑n=0∞[12n+1×e−DE(2n+1)2π2×th2⋅sin(x(2n+1)πh)]}

**Figure 3:**
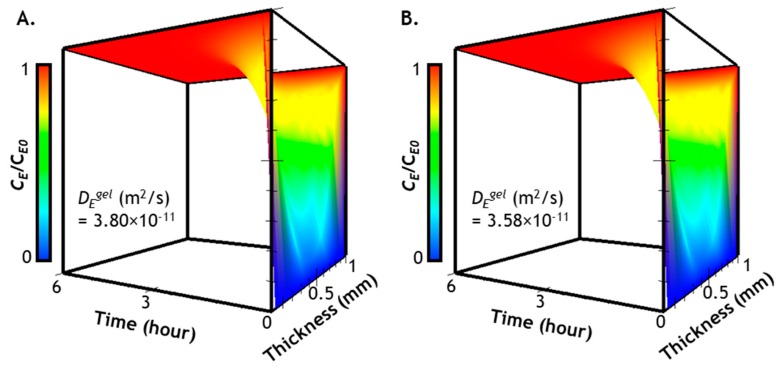
Distribution of *Tyr_ase_* in hydrogels with different crosslinking densities: (**A**) concentration profiles of *Tyr_ase_* in a soft gel (G’ ~0.5 kPa); (**B**) concentration profiles of *Tyr_ase_* in a stiff gel (G’ ~5 kPa). Note that gel thickness was set at 1 mm with the assumption that *Tyr_ase_* diffuses symmetrically from the surfaces (*x* = 0 and *x* = 1) to the center of the hydrogel (*x* = 0.5).

The computational results of Equation (2) represent time- and space-dependent *Tyr_ase_* diffusion into a soft (Figure 3A) or a stiff gel (Figure 3B). We plotted the results from 0 to 6 h, a timeline previously used for *Tyr_ase_*-mediated hydrogel stiffening [27,28]. From the prediction results, it is clear that, regardless of enzyme diffusivity, the entire hydrogel can be equilibrated (i.e., *C_E_/C_E0_* ≈ 1) with the infiltrating enzyme after only 2 h of diffusion. Furthermore, a symmetrical *Tyr_ase_* distribution can be clearly seen along the thickness of the gel owing to the bi-directional diffusion condition. While significant variations of *Tyr_ase_* distribution as a function of time and space are observed in the first 2 h, there is no discernable differences between enzyme diffusion in softer and stiffer hydrogels, suggesting that the stiffening process will not significantly hinder enzyme diffusion in these hydrogels. Finally, a gradient of enzyme concentration can be expected near the surface of the gel within the first 2 h. These predictions have justified that, regardless of gel-network crosslinking density, a period of 6 h is sufficient for *Tyr_ase_*-mediated hydrogel stiffening. 

### 2.4. Verification of Enzyme Diffusion in Non-Stiffening Hydrogels

**Figure 4:**
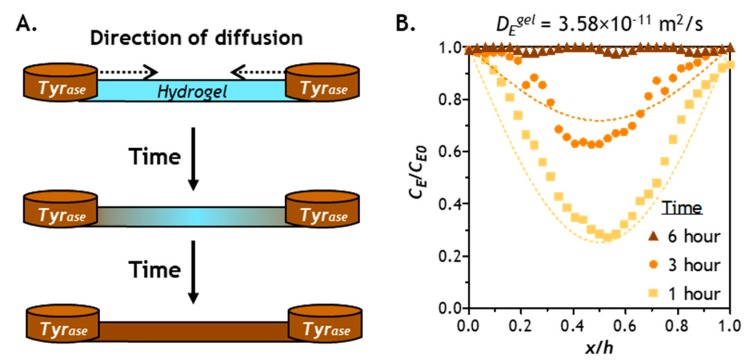
(**A**) Experimental set up for validation of *Tyr_ase_* diffusion into a PEG-peptide (CYGGGYC) hydrogel with 1 mm thickness. (**B**) Comparison of experimental data (symbols) and computational results (dashed lines). Note that the modeling results were derived from the Fick’s second law using diffusivity of *Tyr_ase_* in a stiff gel (G’ ~5 kPa) at 1, 3, and 6 h.

In addition to model predictions, we obtained experimental *Tyr_ase_* diffusion results through imaging *Tyr_ase_* distribution in a hydrogel strip cast in a channel slide connected by two reservoirs filled with solutions containing the enzyme (*C_E0_*). We prepared the hydrogel with high shear moduli (G’ ~5 kPa), which represents a stiffened hydrogel network with the most hindrance to solute transport. Figure 4A illustrates the progression of bi-directional *Tyr_ase_* transport into the thin hydrogel strip (thickness = 1 mm), where *Tyr_ase_* concentration in the hydrogel (*C_E_*) increases as more enzyme molecules infiltrate the hydrogel. Figure 4B shows the experimental *Tyr_ase_* diffusion profiles at 1, 3, and 6 h (i.e., solid symbols), as well as the diffusion model predictions (i.e., dashed lines). After 1 and 3 h of bi-directional diffusion, both experimental data and the model prediction exhibited symmetrical *Tyr_ase_* distribution along the diffusion path. After 6 h of diffusion, *C_E_* at the middle of the gel reached equilibrium with *C_E0_* in both experiment and model prediction. Since the gels were formed at a higher crosslinking density (i.e., G’ ~5 kPa) in this example, a 6-h period should be sufficient for hydrogels with lower stiffness (higher mesh size) to reach equilibrium with *C_E0_*. While these assessments have not yet taken enzyme reactions into account, they complement the diffusion model prediction shown in Figure 3. These results are also supported by experimental stiffening results reported previously, in which gel stiffening was completed within 6 h of *Tyr_ase_* incubation [28].

### 2.5. Effect of Enzyme Concentration on Reaction Velocity

**Figure 5:**
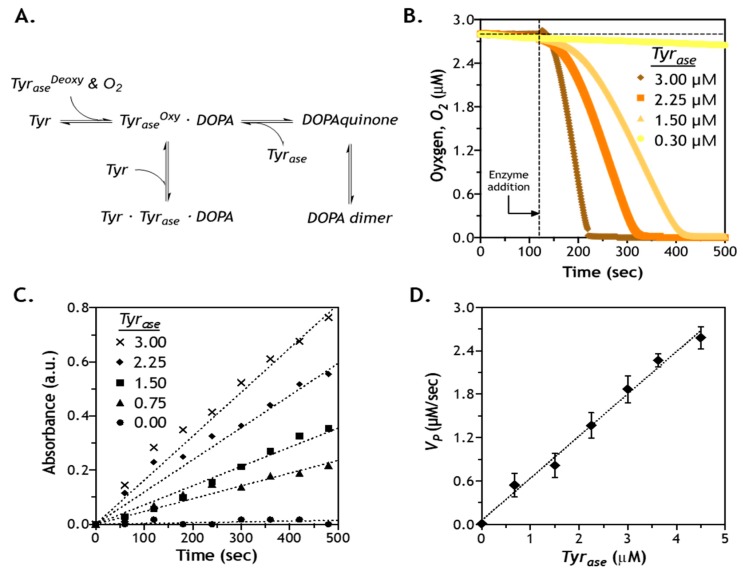
Evaluation of enzymatic reaction kinetics using *Tyr_ase_* and l-Tyr: (**A**) steps of *Tyr_ase_*-catalyzed *DOPA* dimer formation; (**B**) detection of oxygen content as a function of time and enzyme concentration; (**C**) quantification of *DOPA* formation using the MBTH assay (absorbance at 475 nm); (**D**) correlation of reaction velocity (*V_P_*) and *Tyr_ase_* concentration.

In addition to predicting enzyme diffusion in gels with different crosslinking densities, we investigated catalytic reactions of *Tyr_ase_* using different phenolic substrates. *Tyr_ase_* is known to catalyze oxidization of *Tyr*, tyramine, and other phenolic derivatives (e.g., hydroxyphenylacetic acid) into DOPA, DOPAquinone, and finally to DOPA dimers [34]. The catalytic reaction of *Tyr_ase_* involves several steps, including a monophenol cycle, a diphenol cycle, and substrate inhibition (Figure 5A) [37]. First, the non-activated deoxy–tyrosinase (*Tyr_ase_^Deoxy^*) binds with the oxygen molecule (*O*_2_) to generate an activated form of tyrosinase (*Tyr_ase_^Oxy^*). In the presence of l-Tyr, *Tyr_ase_^Oxy^* initiates the monophenol cycle that produces DOPA. Since DOPA and l-Tyr are both substrate of the *Tyr_ase_^Oxy^*, the excess l-Tyr can react with both *Tyr_ase_^Oxy^* and the DOPA–*Tyr_ase_^Oxy^* complexes, and thus reaction may move toward diphenol cycle or substrate inhibition [38]. To gain more insight into these reaction kinetics, we monitored the concentration of dissolved *O*_2_, as its disappearance is the first step in the activation of *Tyr_ase_*. As expected, concentration of dissolved *O*_2_ decreased only after the addition of *Tyr_ase_* (at the 2-min mark) to the l-Tyr containing solution (Figure 5B). *O*_2_ content dropped rapidly, except for the lowest enzyme concentration used (i.e., 0.3 µM). Furthermore, dissolved *O*_2_ was completely depleted within 5, 3.5, and 2 min when the solution was added to 1.5, 2.25, and 3 µM *Tyr_ase_*, respectively. At 0.3 µM *Tyr_ase_*, only ~3% decrease in dissolved *O*_2_ was detected after 6 min of enzyme addition, potentially because the rate of oxygen consumption by the small amount of enzyme was much slower than its replenishment from the air.

Figure 5B suggests that a faster *Tyr_ase_* reaction was accompanied by a rapid consumption of dissolved oxygen. However, the consumption of dissolved oxygen represents only the first step in the *Tyr_ase_* reaction cycle (i.e., from *Tyr_ase_^Deoxy^* to *Tyr_ase_^Oxy^*, Figure 5A). In order to understand the kinetics of subsequent reaction steps, it is necessary to determine the amount of actual product formation. To this end, we utilized MBTH assay to monitor the production of DOPA [39,40]. In principle, MBTH reacts with oxidized substrates through both monophenol and diphenol cycles and produces a visible complex with a pink color [41]. Although higher DOPA contents were detected at higher doses of *Tyr_ase_* (Figure 5C), all reactions (except for 0 µM *Tyr_ase_*) were not completed within 6 min of testing, suggesting that the catalytic step of *Tyr_ase_*/l-Tyr reaction was slower than the rate of oxygen binding to *Tyr_ase_^Deoxy^*. Additionally, the kinetics of DOPA production appeared to be in a linear relationship with respect to time. After plotting reaction velocity as a function of enzyme concentration, a linear correlation was established (Figure 5D). This linear relationship might be due to a relatively high substrate concentration (i.e., 10 mM) when compared with the high binding affinity between l-Tyr and *Tyr_ase_*. In the case of a much smaller *K_M_* compared with *C_S_*, *K_M_* can be omitted in either the regular or the modified Michaelis–Menten equation (see Section 3.5), which yields the following equations:

(3) VP=kcat⋅CE⋅CSKM+CS(1+CSKi)=kcat⋅CE(1+CSKi)

(4) VP=kcat⋅CE⋅CSKM+CS=kcat⋅CE

as shown in Equations (3) and (4), omitting *K_M_* results in a linear correlation between *V_p_* and *C_E_* regardless of the status of substrate inhibition, which can be characterized by a decreasing reaction rate at high substrate concentrations [42]. Using the general or modified Michaelis–Menten equation, we obtained *k_cat_* and *K_M_* for *Tyr_ase_*-mediated reactions (Table 1). Clearly, all three substrates exhibited at least 10-fold lower *K_M_* than the substrate concentration used in the experiments (i.e., 10 mM, Figure 5), thus justifying the omission of *K_M_* in Equations (3) and (4). 

### 2.6. Effect of Substrate Concentration of Enzymatic Reaction

**Figure 6:**
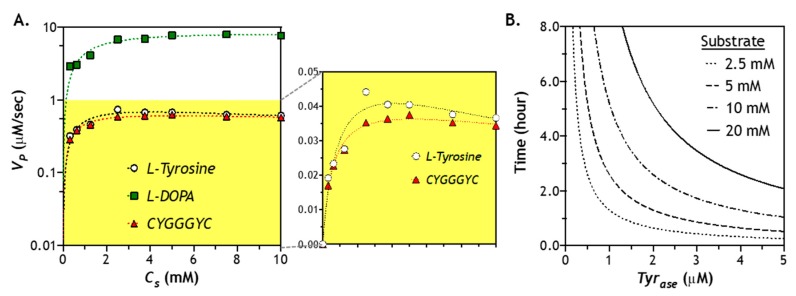
Effect of substrate concentration on *Tyr_ase_*-mediated catalytic reaction: (**A**) reaction kinetics of *Tyr_ase_* (0.6 µM) using different substrates (2–10 mM), including l-Tyr, l-DOPA, and peptide crosslinker (CYGGGYC)—kinetics of l-Tyr and CYGGGYC are highlighted in the right panel; (**B**) estimation of the time for *Tyr_ase_* to convert all substrates into products.

As mentioned earlier, utilization of Tyr for *Tyr_ase_* may exhibit substrate inhibition that reduces catalytic activity of *Tyr_ase_.* To evaluate whether such an effect exists when a peptide substrate is used, we treated a model peptide CYGGGYC with *Tyr_ase_* and used other substrates as controls (e.g., l-Tyr or l-DOPA). As shown in Figure 6A, *Tyr_ase_* exhibited the highest reactivity for l-DOPA (*V_P_* = 8.6 µM/s) among all substrates. The reaction rates for l-Tyr and CYGGGYC were 0.88 and 0.70 µM/s, respectively. *Tyr_ase_*/l-DOPA reaction appeared to proceed through the diphenol cycle without discernable substrate inhibition, as the reaction velocity reached a plateau value at high substrate concentration. Furthermore, we noticed a slightly lower maximum reaction rate at a higher concentration (2–10 mM) of l-Tyr, which was indicative of substrate inhibition (Figure 6A). Therefore, the modified Michaelis–Menten equation (see Section 3.5) was utilized to obtain kinetic constants for *Tyr_ase_*/l-Tyr reaction. Interestingly, there was no significant substrate inhibition when tyrosine-containing peptide (i.e., CYGGGYC) was used as substrate for *Tyr_ase_*. It is likely that the peptidyl tyrosine residues exhibited different affinity (*K_M_*) for *Tyr_ase_*. Indeed, Marumo *et al*. have investigated the effect of peptide sequence on *Tyr_ase_* reaction efficiency [43]. Compared to *Tyr*, some tyrosine-containing peptide sequences (e.g., Gly-Tyr-Gly and Lys-Glu-Thr-Tyr-Ser-Lys) have a higher DOPA conversion ratio when reacting with *Tyr_ase_* due to a higher binding affinity between *Tyr_ase_* and oxidized Tyr. Upon fitting the reaction velocity data with the general or modified Michaelis–Menten model (see Section 3.5), we found that, compared with soluble l-Tyr, peptidyl Tyr exhibited a higher binding affinity (i.e., lower *K_M_*) with *Tyr_ase_* (Table 1). 

Another key aspect in designing an enzymatic stiffening hydrogel is to understand how fast the enzyme fully converts the substrates (e.g., peptidyl tyrosine residues) into products (i.e., additional DOPA dimer crosslinking). At any given enzyme and substrate concentration, the time needed to convert all substrates into products can be approximated through dividing the initial substrate content (*C_S0_*) by the reaction velocity (*V_P_*) and catalytic efficiency (*k_cat_*). Regardless of substrate concentration, there is a hyperbolic relationship between reaction time and enzyme concentration. Naturally, at a higher enzyme concentration, the time needed to convert all substrates (10 mM) into products would be much faster than using a lower enzyme concentration. For example, at 3 µM of *Tyr_ase_*, it would take about 2 h to convert all 10 mM of substrates into products. Without considering the enzyme diffusion, Figure 6B provides a first pass estimation of the time needed to achieve stiffening at any given enzyme and substrate concentration.

### 2.7. Numerical Simulation of Diffusion-Reaction in Hydrogel 

We have separately characterized/analyzed *Tyr_ase_* diffusion (Figure 2, Figure 3 and Figure 4) in hydrogels and studied enzymatic reactions of *Tyr_ase_* with tyrosine-containing peptide crosslinker (Figure 5 and Figure 6). Next, we considered both enzyme diffusion and reaction to obtain profiles of peptide substrate consumption and product formation within the gel network over time and space. First, we generated computational data using *Tyr_ase_* diffusion profile *C_E_*(*x,t*), which served as input for the Michaelis–Menten equation. With experimentally obtained *k_cat_* and *K_M_* for the peptide linker CYGGGYC (Table 1), we employed the Lambert W function to numerically solve the Michaelis–Menten equation (see Section 3.5) [44,45]. First, we derived the substrate consumption rate (Equation (5)) from the general Michaelis–Menten equation:

(5) $$\frac{dC_{S}}{dt}=-\frac{V_{Max}\times C_{S}}{K_{M}+C_{S}}\Rightarrow K_{M}\times \ln(\frac{C_{S}{}_{0}}{C_{S}})+(C_{S}{}_{0}-C_{S})=V_{Max}\times t$$

where the space- and time-dependent *C_S_*(*x,t*) can be expressed as:

(6) $$C_{S}(x,t)=K_{M}\times W\left\{f(x,t)\right\}$$

The space- and time-dependent product (i.e., oxidized tyrosine or *DOPA*) concentration can be expressed using the following equation, since it is equivalent to the amount of substrate (i.e., tyrosine) consumed:

(7) $$C_{P}(x,t)=C_{S0}-K_{M}\cdot W\left\{f(x,t)\right\}$$

(8) ln{f(x,t)}=W{f(x,t)}+ln{W{f(x,t)}}

(9) f(x,t)=CS0KM⋅e(CS0−CE(x,t)⋅kcat⋅tKM)

**Figure 7:**
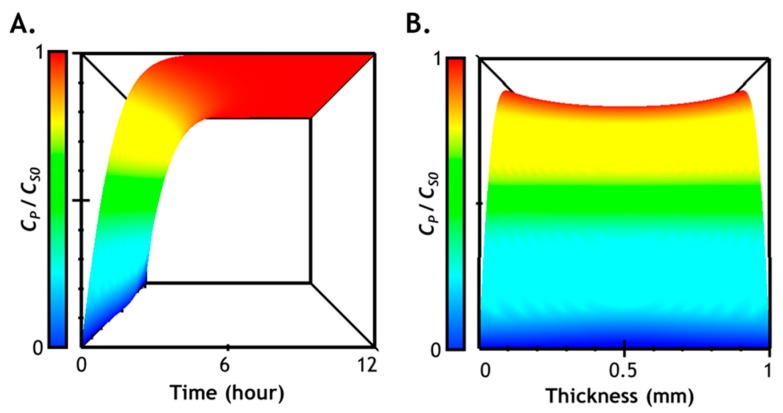
Prediction of time- and space-dependent enzyme-catalyzed product formation in a hydrogel: (**A**) viewing from the *y*-axis (time) in the 3D chart (time, space, and *C_P_*); (**B**) viewing from the *x*-axis (space) in the 3D chart. *D_E_^gel^* = 3.58 × 10^−11^ m/cm^2^, *Tyr_ase_* = 3 µM.

We plotted solution of Equation (7) in Figure 7, which describes the formation of DOPA dimers owing to the *Tyr_ase_*-mediated reaction within the gel network. The prediction results demonstrate that the majority of network-immobilized tyrosine residues will be converted to product within the first 6 to 8 h of *Tyr_ase_* (3 µM) diffusion/reaction. Furthermore, comparing simulation results shown in Figure 3 (i.e., enzyme distribution in hydrogel) with Figure 7 (product formation in hydrogel), it is clear that, while enzyme diffusion may not be critically affected during the stiffening process (Figure 3), the rate of product formation lags behind enzyme diffusion. Hence, it is critically important to take both diffusion and reaction into account when predicting the degree of additional crosslink formation. 

### 2.8. Correlation of Hydrogel Mechanical Property and Its Microstructure

**Figure 8:**
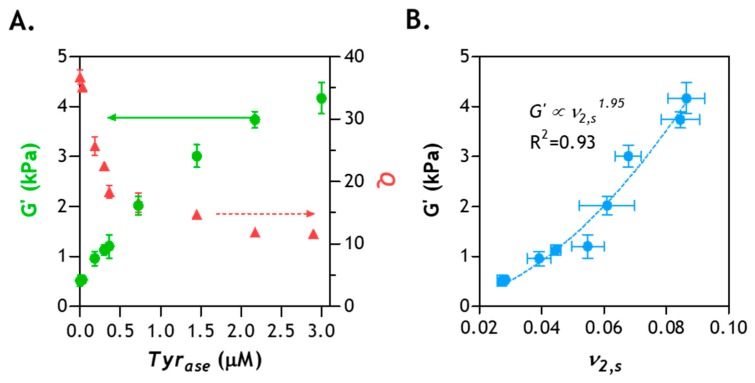
Correlation of hydrogel physical properties and enzyme concentration: (**A**) shear moduli (*G’*) and volumetric swelling ratio (*Q*) of hydrogel stiffened by different concentrations of *Tyr_ase_*—all gels were initially crosslinked by thiol–norbornene photopolymerization using 2.5 wt% PEG8NB, 5 mM peptide crosslinker (CYGGGYC), and 1 mM photoinitiator (LAP); (**B**) correlation of the polymer volume fraction (*v_2,s_*) with *G’* (*N* = 3, Mean ± SEM).

In our previous experimental results, we have shown that a period of 6 h is sufficient to induce enzymatic hydrogel stiffening [27,28]. Further increasing the enzyme incubation time to 12–48 h only marginally increased gel stiffness. Prior experimental observations were largely in agreement with the diffusion-reaction modeling results (Figure 7). To gain a deeper understanding of the crosslinking of enzyme-stiffened hydrogels, we prepared additional PEG8NB–CYGGGYC hydrogels (*G_0_^’^* ~1 kPa) and performed enzyme-induced stiffening using different concentrations of *Tyr_ase_* (0–3 µM). After 6 h of stiffening (and overnight washing to remove residue enzyme), gel moduli and swelling ratios were measured. As shown in Figure 8A, *Tyr_ase_* treatment led to increased gel shear moduli (from ~1 to ~4 kPa) and decreased volumetric swelling ratio (from ~36 to ~12), a correlation commonly observed in chemically crosslinked hydrogels [14,46]. Next, we examined whether the correlation of *G*’ and the polymer volume fraction (*ν_2,s_*, a reciprocal of *Q*) of the enzyme-stiffened hydrogels could be described by the classical rubber elasticity theory, which predicts a linear dependency between *G’* and *ν_2,s_* [10,47]. As shown in Figure 8B, the correlation of experimentally obtained *G’* and *ν_2,s_* follows a power law with an exponent of 1.95. This value is significantly higher than the linear dependence (i.e., exponent = 1) predicted by the theory of rubber elasticity, which suggests the presence of network non-ideality caused either by ineffective initial crosslinking (performed with low macromer concentration) [16] and/or enzymatic stiffening. However, it should be noted that this degree of network non-ideality (i.e., the exponent in the power law) is similar to other chemically crosslinked hydrogels [48]. Therefore, it can be concluded that the structure-function relationships of enzyme-stiffened hydrogels are similar to other chemically crosslinked hydrogels.

In summary, we have predicted and validated enzyme diffusion in gels with varying stiffness (Figure 2, Figure 3 and Figure 4). We have also monitored *Tyr_ase_*-mediated reactions and predicted the degree of gel stiffening from the perspective of product formation under varying enzyme and substrate concentrations (Figure 5 and Figure 6). The modeling and experimental results have demonstrated that while a period of 3 h is sufficient for the enzyme to equilibrate the entire gel, it takes at least another 3 h for the reactions to be completed (Figure 7) [28]. Furthermore, the enzyme-stiffened hydrogels exhibit physical properties (e.g., swelling and modulus) similar to other chemically crosslinked hydrogels (Figure 8). Collectively, the information acquired from this study should be highly useful in designing enzyme-mediated dynamic hydrogel system for fundamental material science and tissue engineering applications.

## 3. Materials and Methods

### 3.1. Materials

Hydroxyl-terminated 8-arm PEG (20 kDa) and 5-norbornene-2-carboxylic acid were purchased from JenKem Technology (Plano, TX, USA) and Sigma-Aldrich (St. Louis, MO, USA), respectively. All reagents and Fmoc-capped amino acids for solid-phase peptide synthesis were acquired from Anaspec (Fremont, CA, USA) or ChemPep (Wellington, FL, USA). Other reagents for chemical synthesis were purchased from Sigma-Aldrich or Thermo Fisher (Waltham, MA, USA) unless noted otherwise. 

### 3.2. Macromer Preparation and Peptide Synthesis

PEG8NB and lithium phenyl-2,4,6-trimethylbenzoylphosphinate (LAP) were synthesized as described elsewhere [16,49,50]. Peptides (e.g., CYGGGYC) were synthesized via standard Fmoc coupling chemistry on an automated, microwave-assisted peptide synthesizer (Liberty 1, CEM, Matthews, NC, USA). The crude products were cleaved in a trifluoroacetic acid (TFA) cleavage cocktail composed of 7.6 mL trifluoroacetic acid (TFA), 0.2 mL triisopropylsilane (TIPS), 0.2 mL distilled water, and 400 mg phenol. The cleaved and dried peptides were purified by reverse-phase HPLC (PerkinElmer Flexar system) using 95%/5% (*v*/*v*) water/acetonitrile (ACN) with a trace amount (0.1 vol %) of TFA as the starting mobile phase. A linear gradient of ACN was employed to separate the products through a semi-prep peptide C18 column (5 mL/min). The separated products were monitored with a UV/vis detector and the purified peptides were characterized with liquid chromatography coupled with mass spectrometry (1200 series LC/MS system, Santa Clara, CA, USA). 

### 3.3. Modeling of Enzyme Diffusion into Hydrogels

We used Fick’s second law of diffusion (Equation (10)) to estimate distribution of *Tyr_ase_* in hydrogel over space and time:

(10) $$\frac{\partial C_{E}(x,t)}{\partial t}=-\frac{\partial }{\partial x}(D_{E}{}^{gel}(x,t)\times \frac{\partial C_{E}(x,t)}{\partial x})$$

Note that the diffusivity of the enzyme in hydrogel (*D_E_^gel^*) negatively scales with hydrogel crosslinking density and positively correlates to hydrogel mesh size (*ξ*). As enzyme infiltrates the hydrogel, it catalyzes tyrosine residues into DOPA dimers, resulting in an increase in hydrogel crosslinking density (i.e., smaller mesh size) that may lead to lower diffusivity. Hence, *D_E_^gel^* may be dependent on the location of the enzyme in the hydrogel. In this mathematical model, we assume that enzyme diffusion proceeds bi-directionally from the top and bottom (i.e., along the *x*-axis) of the thin hydrogel with a thickness of *h* (i.e., neglecting diffusion from the gel edge). Initially, the entire hydrogel (i.e., 0 < *x* < *h*) is free of enzyme, which gives the following initial condition:

(11) $$C_{E}(x,0)=0$$

we also assume that enzyme concentration in the solution remains unchanged (*C_E0_*), which yields the following boundary conditions:

(12) $$\begin{array}{l} C_{E}(0,t)=C_{E0}\\ C_{E}(h,t)=C_{E0} \end{array}$$

since enzyme diffusion proceeds bi-directionally, at any given time there is no flux at the center of the hydrogel (i.e., *x* = *h*/2), which yields the following boundary condition:

(13) $$\frac{\partial C_{E}\left(\frac{h}{2},t\right)}{\partial t}=0$$

As mentioned earlier, diffusivity of any solute in a crosslinked hydrogel is affected by gel mesh size. Hence, it is necessary to determine whether the additional DOPA dimer crosslinks significantly affects *Tyr_ase_* transport in the stiffened gel. In this regard, the Lustig–Peppas estimation of solute diffusivity in a highly swollen gel can be used to establish a correlation between *Tyr_ase_* diffusivity and hydrogel mesh size [35].

(14) DEgel(x,t)=DEsol⋅(1−REξ)⋅e−Y(Q−1)

where *D_E_^sol^* is the diffusivity of enzyme in solution (5.05 × 10^−10^ m^2^/s for *Tyr_ase_*), *R_E_* is the hydrodynamic radius of the enzyme (4.5 nm for *Tyr_ase_*) [51], and *Y* is the critical volume required for a successful translational movement of the substrate relative to the average free volume of a water molecule (1 for PEG-based gels) [35]. Note that this equation is used only when gel mesh size is larger than hydrodynamic radius of the soluble molecule (i.e., *R_E_*/*ξ* < 1) because no diffusion is possible when R_E_ is larger than *ξ*. Clearly, when *R_E_* approaches *ξ*, *D_E_^gel^* becomes much smaller than *D_E_^solution^*. Therefore, it is critical to determine the relative sizes of *R_E_* and *ξ* even for highly swollen PEG-based hydrogels (i.e., *Q* > 10). To obtain mesh size of a swollen hydrogel, we first obtained mass swelling ratio (*q*) by measuring the mass (*m*) of swollen and dried gels:

(15) $$q=\frac{m_{Swollen\;gel}}{m_{Dried\;gel}}$$

where the volumetric swelling ratio (*Q*) of the hydrogel is determined using mass swelling ratio (*q*) and the density (*ρ*) of PEG (1.087 g/cm^3^ at 37 °C) and water (0.994 g/cm^3^ at 37 °C):

(16) $$Q=\frac{\rho_{H_{2}O}}{(q-1)\rho_{H_{2}O}+\rho_{PEG}}$$

once *Q* is obtained, gel mesh size (*ξ*) can be derived using the following equation:

(17) $$\xi =Q^{\frac{1}{3}}\cdot \ell \cdot (\frac{3C_{n}\bar{M_{c}}}{\bar{M_{n}}})$$

where ℓ is the average bond length (1.47 Å) in the backbone of an ethylene glycol subunit (i.e., –CH_2_–CH_2_–O–), 3 is the total number of bonds in a PEG repeat subunit, *C_n_* is the Flory characteristic ratio (4 for PEG-based hydrogel), and $\bar{M_{n}}$ is the number for average molecular weight of PEG8NB (20 kDa) [1]. To obtain the average molecular weight between crosslinks ($\bar{M_{c}}$) within a step-growth hydrogel, the following equation can be used:

(18) $$\bar{M_{c}}=2(\frac{MW_{A}}{f_{A}}+\frac{MW_{B}}{f_{B}})$$

where *MW_A_* and *MW_B_* represent molecular weights of the two macromer crosslinkers PEG8NB and CYGGGYC, respectively. *f_A_* and *f_B_* are the functionality of the macromer and peptide crosslinker (i.e., 8 and 2). Numerical model predictions were programmed and executed via Grapher (Arizona Software) using a spatial step size (Δ*x*) of 1 µm and a temporal step size (Δ*t*) of 1 s.

### 3.4. Characterization of Oxygen Consumption

During the oxidation of Tyr, *Tyr_ase_* exhibits four distinct oxidation states (*deoxy*-, *oxy*-, *met*-, and *deact-Tyr_ase_*), and the oxygen molecule (*O_2_*) is a key component initiating the overall catalytic processes [39]. The association of *O_2_* and *Tyr_ase_* forms oxy-*Tyr_ase_* that oxidizes Tyr (or other phenolic precursors) into DOPAquinone. Hence, the consumption of soluble *O*_2_ can be used to gauge the extent of *Tyr_ase_*-catalyzed DOPA formation. To this end, we measured soluble *O*_2_ contents in a 2 mL microtube which contained l-Tyr (10 mM) and *Tyr_ase_* (0.1, 1.5, and 3 µM) by a portable fiber optic oxygen probe and meter (PreSens Microx 4, Regensburg, Germany). The needle-type probe was extended into the center of the solution. Note that the *O*_2_ quantifications were started 2 min prior to *Tyr_ase_* addition.

### 3.5. Tyr_ase_-Mediated Reaction Kinetics 

The kinetics of *Tyr_ase_*-mediated reactions were modeled using standard Michaelis–Menten equation [52]:

(19) $$-V_{s}=V_{P}=\frac{V_{max}\cdot C_{S}}{K_{M}+C_{S}}=\frac{C_{E}\cdot k_{cat}\cdot C_{S}}{K_{M}+C_{S}}$$

where *C_S_* and *C_P_* are the concentration of substrate and product, respectively; *V_S_* and *V_P_* are the velocity of substrate consumption and product formation; and *V_max_* is the maximum reaction velocity, which is equivalent to enzyme concentration (*C_E_*) multiplied by the turnover number (*k_cat_*). 

In the event of substrate inhibition, a modified Michaelis–Menten equation was employed [53]:

(20) $$V_{P}=\frac{C_{E}\cdot k_{cat}\cdot C_{S}}{K_{M}+C_{S}(1+\frac{C_{S}}{K_{i}})}$$

here, *K_i_* is the kinetic constant for substrate inhibition [38]. 

We utilized 3-Methyl-2-benzothiazolinone hydrazone (MBTH, 2 mM in pH 6.5 PBS) as an indicator to characterize enzymatic reactions between *Tyr_ase_* (0.6 µM) and various substrates (0–10 mM), including l-Tyr, l-DOPA, and CYGGGYC. MBTH is a hydrazone-based compound that complexes with DOPAquinone to produce a pink pigment, which can be quantified via measuring absorbance at 475 nm [40,41]. Note that cysteine residue also reacts with MBTH. Hence, prior to the MBTH assay, all peptides were conjugated with linear norbornene-functionalized PEG (PEGdNB) through thiol–norbornene photo-click reaction (1 mM LAP, 365 nm light at 5 mW/cm^2^, 2 min). The enzymatic reactions were allowed to proceed for 20 min and the increases in 475 nm absorbance were monitored with a microplate reader (Biotek, Synergy HTX, Winooski, VT, USA). At any given *Cs*, reaction velocity (*V_P_*) can be obtained by calculating the slope of absorbance vs. the time curve. Next, *V_Max_* and *Cs* values were fitted to the general or modified Michaelis–Menten model (i.e., Equation (19) or (20)) to acquire the *k_cat_* and Michaelis constant (*K_M_*). Additionally, to understand the correlation between *C_E_* and *V_P_*, we also treated the substrate (l-Tyr, 10 mM) with varying *Tyr_ase_* concentrations (0–3 µM). The reactions were monitored via the MBTH assay as described above. 

### 3.6. Fabrication and Characterization of the Step-Growth PEG-Peptide Hydrogels

PEG-peptide hydrogels were prepared by coupling norbornene moieties of PEG8NB and the terminal cysteine moieties of CYGGGYC via thiol–norbornene photopolymerization. Hydrogels were prepared with different macromer contents but with the stoichiometric ratio of thiol to norbornene maintained at 1 to minimize network defects. In brief, aliquots of precursor solutions (45 µL) were deposited between two glass slides separated by silicon spacers (1 mm). Hydrogel crosslinking was initiated by 365 nm light (5 mW/cm^2^) exposure for 2 min. Following the initial photopolymerization, gels were maintained in PBS at 37 °C for 24 h prior to characterization or *Tyr_ase_*-mediated stiffening. Shear moduli (strain-sweep mode) of the pre- and post-stiffened hydrogels were characterized using a digital rheometer (Bohlin CVO 100, Malvern Instruments, Malvern, Worcestershire, UK) equipped with an 8-mm diameter parallel plate geometry. Measurements of gel shear moduli were acquired from the average of the linear region on the modulus-strain curve (0.1–5% strain) with oscillation frequency set at 1 Hz. 

### 3.7. Statistical Analysis

All experiments were repeated three times independently with four samples per condition. Experimental results were reported as Mean ± SEM with a sample size of at least three (*n* = 3). The data were analyzed by one-way or two-way ANOVA with GraphPad Prism 8 software. Single, double, and triple asterisks represent *p* < 0.05, 0.001, and 0.0001, respectively, and *p* < 0.05 is considered statistically significant. 

## Figures and Tables

**Table 1:** Kinetic constants for *Tyr_ase_*-mediated reactions.

	l-Tyrosine	l-DOPA	CYGGGYC
*k_cat_ (s^−1^)*	0.93	8.63	0.60
*K_M_ (mM)*	0.85	1.02	0.58
*K_i_ (mM)*	19.85	-	-
*R^2^*	0.956	0.971	0.98
